# Mitigating the Impact of Long-Term Construction on the Health of Older Adult Residents in New York City’s Chinatown

**DOI:** 10.1093/geroni/igaa057.090

**Published:** 2020-12-16

**Authors:** Simona Kwon, Yi-Ling Tan, Jennifer Wong, Janet Pan

**Affiliations:** NYU School of Medicine, New York, New York, United States

## Abstract

Introduction: Recent proposed major construction projects in New York City’s Chinatown often last multiple years. Little is known about the health impact of construction on vulnerable populations such as older adults. In Chinatown, approximately 20% of residents are older adults, live below the poverty level (34%), have a disability (47%), and nearly half report limited English proficiency. Objectives: We are conducting a mixed methods study to describe possible health and psychosocial outcomes of construction on older adults in Chinatown. Methods: We used a community-engaged modified Delphi process to identify priority areas related to construction and older adults which included: 1) a scoping review of the health impact of long-term construction; 2) key informant interviews of academic experts; and 3) convened community stakeholder leaders to review key focus areas and evidence-informed, culturally-relevant mitigation strategies. Five priority topics were identified: 1) Construction site emissions; 2) Noise; 3) Outdoor nocturnal lighting; 4) Neighborhood changes; and 5) Relocation. Results: Long-term construction contributes to adverse effects of air pollution, noise, and changes in the environment, with exposure to particulate matter and unwanted noise associated with higher morbidity and mortality. Unsafe sidewalk due to construction increase the risk of falling, the leading cause of death among NYC seniors. Construction-related stressors may isolate older adults from vital services and social networks. Conclusion: Long-term construction poses serious health implications for older adults. Stakeholders should adopt a community-engaged approach and identify meaningful community priorities to inform practical solutions to mitigate the impact of construction on vulnerable Chinatown older adults.

